# Comparative Study of Adalimumab, Infliximab and Certolizumab Pegol in the Treatment of Cystoid Macular Edema Due to Behçet’s Disease †

**DOI:** 10.3390/jcm13237388

**Published:** 2024-12-04

**Authors:** Nuria Barroso-García, José Luis Martín-Varillas, Iván Ferraz-Amaro, Lara Sánchez-Bilbao, Adrián Martín-Gutiérrez, Alfredo Adán, Inés Hernanz-Rodríguez, Emma Beltrán-Catalán, Miguel Cordero-Coma, David Díaz-Valle, Marisa Hernández-Garfella, Lucía Martínez-Costa, Manuel Díaz-Llopis, José M. Herreras, Olga Maíz-Alonso, Ignacio Torre-Salaberri, Antonio Atanes-Sandoval, Santos Insúa, Raquel Almodóvar-González, Patricia Fanlo, Juan Ramón De Dios Aberasturi, Ángel García-Aparicio, Sergio Rodríguez-Montero, Vega Jovaní, Patricia Moya-Alvarado, Eva Peña Sainz-Pardo, Vanesa Calvo-Río, Rosalía Demetrio-Pablo, José Luis Hernández, Ricardo Blanco

**Affiliations:** 1 Rheumatology, Hospital Regional Universitario, Universidad de Málaga (UMA), 29010 Málaga, Spain; nubarroso@hotmail.com; 2 Rheumatology, Hospital de Laredo, Instituto de Investigación Valdecilla (IDIVAL), 39770 Cantabria, Spain; jlmvarillas@gmail.com; 3 Immunopathology Group, Marqués de Valdecilla University Hospital, Instituto de Investigación Valdecilla (IDIVAL), 39011 Santander, Spainhernandezjluis@gmail.com (J.L.H.); 4 Rheumatology, Hospital Universitario de Canarias, 38320 Santa Cruz de Tenerife, Spain; iferrazamaro@hotmail.com; 5 Rheumatology, Ophthalmology and Internal Medicine, Hospital Universitario Marqués de Valdecilla, Instituto de Investigación Valdecilla (IDIVAL), 39008 Santander, Spain; 6 Ophthalmology, Hospital Clinic de Barcelona, 08036 Barcelona, Spain; 7 Rheumatology, Hospital del Mar, 39008 Barcelona, Spain; 8 Ophthalmology, HM Hospitales & Hospital Regional Universitario, 29010 Málaga, Spain; 9 Ophthalmology, Hospital Clínico San Carlos, 28040 Madrid, Spain; 10 Ophthalmology, Hospital Universitario General Valencia, 46014 Valencia, Spain; 11 Ophthalmology, Hospital Universitario Doctor Peset, 46017 Valencia, Spain; 12 Ophthalmology, Hospital Universitario La Fe, 46026 Valencia, Spain; 13 Ophthalmology, Hospital Clínico Universitario de Valladolid, 47003 Valladolid, Spain; 14 Rheumatology, Hospital Universitario de Donosti, 20014 San Sebastián, Spain; 15 Rheumatology, Hospital Universitario de Basurto, 48013 Barakaldo, Spain; 16 Rheumatology, Complejo Hospitalario Universitario de A Coruña, 15006 A Coruña, Spain; 17 Rheumatology, Hospital Universitario de Santiago de Compostela, 15706 A Coruña, Spain; 18 Rheumatology, Hospital Universitario Fundación de Alcorcón, 28922 Madrid, Spain; 19 Internal Medicine, Complejo Hospitalario Universitario de Navarra, 31008 Navarra, Spain; 20 Rheumatology, Hospital Universitario de Álava, 01009 Álava, Spain; 21 Rheumatology, Hospital Universitario de Móstoles, 28935 Madrid, Spain; 22 Rheumatology, Hospital Universitario Virgen de Valme, 41014 Sevilla, Spain; 23 Rheumatology, Hospital General Universitario de Alicante, 03010 Alicante, Spain; 24 Rheumatology, Hospital de la Santa Creu i Sant Pau, 08041 Barcelona, Spain; 25 Pediatric, Hospital Universitario La Paz, 28046 Madrid, Spain

**Keywords:** uveitis, cystoid macular edema, Behçet disease, TNF inhibitor monoclonal antibodies, adalimumab, infliximab, certolizumab pegol

## Abstract

**Background:** The leading cause of blindness due to non-infectious uveitis is cystoid macular edema (CME). Behçet’s disease (BD) is one of the most commonly conditions related to CME. **Objectives:** To compare the effectiveness and safety of adalimumab (ADA), infliximab (IFX) and certolizumab (CZP) in refractory CME due to BD. **Methods**: Multicenter study of BD-CME patients with no response to glucocorticoids (GCs) and at least one conventional immunosuppressive drug. At baseline, all patients presented CME, defined by OCT > 300 µ. The effectiveness of ADA, IFX and CZP was assessed over a 2-year period from baseline using the following ocular parameters: macular thickness (µm), visual acuity (BCVA), anterior chamber (AC) cells and vitritis. Mixed-effects regression models were applied. **Results:** a total of 50 patients (75 eyes) were studied (ADA = 25; IFX = 15 and CZP = 10). No significant differences in demographic parameters were found among the three groups. However, individuals in the CZP group had a significantly extended time from diagnosis to treatment onset (72 (36–120) months, *p* = 0.03) and had received a higher number of biological therapies (1.7 ± 1.1) compared to the ADA and IFX groups. Within the CZP group, ADA and IFX were previously administrated in seven patients. After 2 years of follow-up, a rapid and sustained reduction in macular thickness was noted in all three groups with no significant differences between them. Additionally, enhancements in BCVA, AC cells and vitritis were also observed. No serious adverse events were reported in the CZP group, although one isolated case of bacteremia was documented in the ADA group. ADA, IFX and CZP appear to be effective and safe treatments for refractory CME in BD. CZP seems to remain effective even in patients with an insufficient response to ADA and/or IFX. **Conclusions:** ADA, IFX and CZP appear to be effective and safe treatments for refractory CME in BD. CZP seems to remain effective even in patients with an insufficient response to ADA and/or IFX.

## 1. Introduction

Behçet’s disease (BD) is classified, according to the Chapel Hill consensus, as a variable vessel vasculitis due to the involvement of arteries and veins of any size [1]. Therefore, BD is characterized by the heterogeneity of its clinical manifestations, with mucocutaneous lesions and ocular involvement being the most common presentation [2]. Ocular manifestations, particularly uveitis, are some of the most severe and occur in approximately 70% of patients, leading to visual loss in 13–74% of them [3,4,5,6]. The main cause of visual acuity impairment in BD uveitis is the formation of cystoid macular edema (CME), and it appears in about one-third of patients [7]. CME is characterized by a retinal thickening in the macular area due to the breakdown of the blood–retina barrier and the accumulation of extracellular fluid in the intraretinal or subretinal space [8]. Proinflammatory conditions in BD and increased intraocular cytokines such as tumor necrosis factor (TNF-alpha), interleukin-6 (IL-6) or interleukin-17A (IL-17A) lead to endothelial permeability and induce the production of vascular endothelial growth factor (VEGF) [8,9,10,11,12,13].

The treatment of CME should be performed early, with high-dose glucocorticoids and conventional immunosuppressants (ISs). In the last 2 decades, the prognosis of patients with visual damage refractory to conventional therapy due to BD has improved dramatically thanks to the use of biologic drugs, especially anti-TNF-alpha drugs [14]. Adalimumab (ADA) and infliximab (IFX) are the most widely studied drugs. Based on the Phase 3 clinical trials “VISUAL I, II and III”, ADA is the only drug approved by the FDA and EMA for the treatment of non-infectious non-anterior uveitis [15,16,17]. IFX is authorized in Japan (Pharmaceuticals and Medical Devices Agency, PMDA) for use in uveoretinitis due to BD [18,19]. In addition, both drugs have been extensively studied in real clinical practice, demonstrating their effectiveness and good safety profile [20,21,22,23,24,25,26]. However, other anti-TNF-alpha agents such as certolizumab pegol (CZP) have limited evidence for ocular involvement in BD patients, with data from small case series or heterogeneous populations with different diseases focused in spondyloarthritis [27,28,29,30].

Taking all these considerations into account, we aimed to compare the effectiveness of ADA, IFX and CZP in patients with refractory CME due to BD uveitis.

## 2. Patients and Methods

### 2.1. Design, Enrolment Criteria and Definitions

We conducted a national multicenter observational study of 50 BD patients with refractory CME treated with anti-TNF-alfa drugs (ADA, IFX and CZP). We used a cohort of 177 patients with BD uveitis and we selected only those with CME refractory to corticosteroids and at least one conventional IS at baseline. In addition, all received anti-TNF-alpha drugs at baseline: 25 patients were treated with ADA, 15 patients with IFX [26] and CZP was administered in the remaining 10 patients [29]. The conventional IS drugs and dosages used before anti-TNF-alfa therapy were as follows: azathioprine (AZA) 100–150 mg/day orally, cyclosporine A (CsA) 3–6 mg/Kg/day orally, Methotrexate (MTX) 7.5–25 mg/week orally or orally and Mycophenolate Mofetil (MMF) 2–3 g/day orally. In the case of severe CME, the therapeutic scheme included three consecutive pulses of methylprednisolone (MP), 500–1000 mg/day.

ADA was administered at the standard dose of 40 mg/2 weeks subcutaneously (SC) with or without a loading dose (80 mg); IFX 3–5 mg/kg intravenously (IV) at 0, 2 and 6 weeks and then every 4–8 weeks; and CZP was prescribed at the standard dose of 400 mg at baseline and weeks 2 and 4, and then continued as 200 mg injections every other week.

BD was diagnosed according to International Study Group (ISN) classification criteria and/or to the International revised Criteria for BD (ICBD) [31,32].

Before anti-TNF-alfa treatment, the presence of infectious diseases or malignancy was excluded. A chest radiograph and a tuberculin skin test (TST) and/or an interferon-g assay (quanti-FERON, TB Gold Plus (QTF-Plus)) were performed to exclude latent tuberculosis. If tuberculosis was detected, prophylactic treatment with isoniazid was prescribed for at least 4 weeks before treatment and maintained for 9 months.

The anatomical classification of uveitis was performed according to the Standardization of Uveitis Nomenclature (SUN) Working Group [33].

Remission was defined as the absence of intraocular inflammation for at least 3 months [34].

Written consent was obtained from all patients who received treatment with IFX and CZP as off-label drugs by the EMA for the treatment of non-infectious non-anterior uveitis. In addition, the corresponding ethics committee approval was obtained (CEIm 2023.439).

### 2.2. Outcome Variables

To determine effectiveness, macular thickness was the main variable studied. Other evaluated variables were intraocular inflammation (anterior chamber cells and vitritis), BCVA, the presence of retinal vasculitis and glucocorticoids’ sparing effect. These outcomes were recorded at baseline (ADA, IFX and CZP initiation) and in the 1st week, 1st month, 3rd month, 6th month and 1st and 2nd years. To assess the macular thickness, High-Definition Optical Coherence Tomography (OCT), using a Cirrus HD-OCT (Carl Zeiss, Dublin, CA, USA), was performed. Scans were obtained using the 512 × 128 scan pattern. Macular thickening was defined as a macular thickness >250 µm. CME was established as macular thickening >300.

The degree of intraocular inflammation was evaluated according to the SUN Working Group [33,34]. Vitritis was assessed by the Nussenblatt scale [34].

The best-corrected visual acuity (BCVA) was estimated using the Snellen chart. For the present study, 20/20 (normal vision) was expressed as 1.0 and 0/20 as 0.0 [35].

To evaluate the presence of retinal vasculitis, fluorescein angiography (FA) was performed. Retinal vasculitis was defined as retinal angiographic leakage, staining and/or occlusion on FA [6].

### 2.3. Statistical Analysis

The demographic and clinical characteristics of each group of patients (ADA, IFX and CZP groups) were described as mean ± standard deviation (SD) or percentages for categorical variables. For non-normally distributed continuous variables, data were expressed as a median and interquartile range (IQR). Univariate differences between patients in the 3 treatment categories were assessed through ANOVA or Kruskal–Wallis tests according to normal distribution or the number of subjects. To test whether differences were observed between the three treatments after 2 years of follow-up, we performed a linear mixed models analysis with repeated measures and an intergroup factor (three treatments). “*p* values” < 0.05 were considered as statistically significant. Statistical analysis was performed using Stata software, version 17/SE (Stata Corp., College Station, TX, USA) [36].

## 3. Results

### 3.1. Baseline Demographic and Clinical Features of the Study Sample

Fifty BD patients (75 affected eyes) with refractory CME to conventional treatment who received ADA (*n* = 25, 40 affected eyes), IFX (*n* = 15, 21 affected eyes) and CZP (*n* = 10, 14 affected eyes) were included in this study. Table 1 summarizes the main baseline characteristics. No statistically significant differences were found in the mean age of the patients, the predominance of gender or the prevalence of HLA-B51, although in the group of patients with CZP, there was a slight predominance of female sex and a lower presence of HLA-B51.

However, there were baseline differences between the three groups in the time from uveitis appearance to anti-TNF-alfa therapy initiation: 27 (12–60) months in the ADA group, 15 (8–60) months in the IFX group and 72 (36–120) months in the CZP group (*p* = 0.03).

In the three groups, no statistically significant differences were observed in baseline macular thickness. Regarding ocular inflammation parameters, patients treated with ADA presented with significantly higher vitritis values compared to the other two groups (2.75 (1.75–3) ADA, 1 (0–2) IFX and 1 (0–2) CZP; *p* = 0.02) (Table 1).

Concerning prior conventional IS received before anti-TNF-alpha drugs, no significant differences were observed between three groups. Patients in the ADA and IFX groups did not receive prior biologic therapy. Nevertheless, patients in the CZP group were treated with at least one prior anti-TNF-alpha drug, with a mean of 1.7 ± 1.1 drug per patient (ADA *n* = 7, IFX *n* = 7, golimumab *n* = 2 and etanercept *n* = 1). In two patients, CZP was initiated as the first anti-TNF-alpha agent (Table 1). The CZP group received a lower dose of prednisone than the other two groups at the start of biologic treatment. Relative to the median dose of prednisone, the CZP group received a lower dose (9 (6–20) mg) than the ADA and IFX groups (45 (25–60) and 30 (20–60) mg, respectively; *p* = 0.051) at the start of biologic treatment but with no statistical significance.

### 3.2. Visual Outcome and Follow-Up

**Figure 1:**
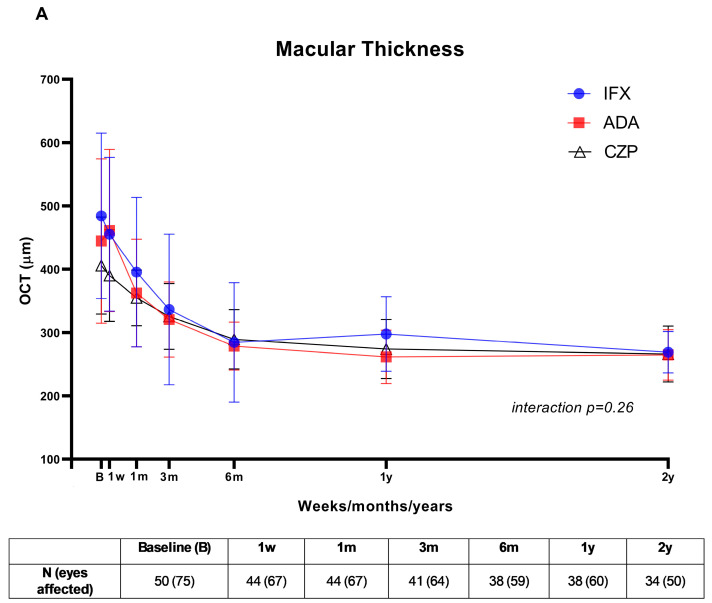

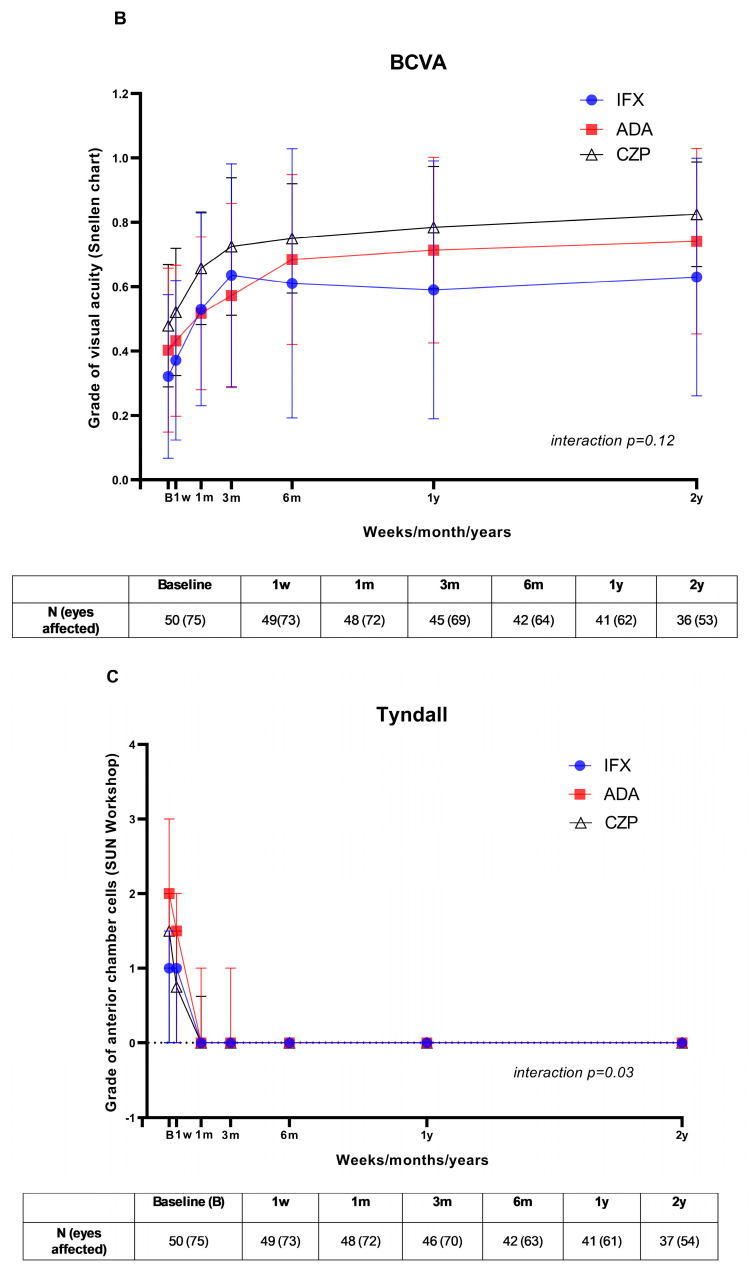

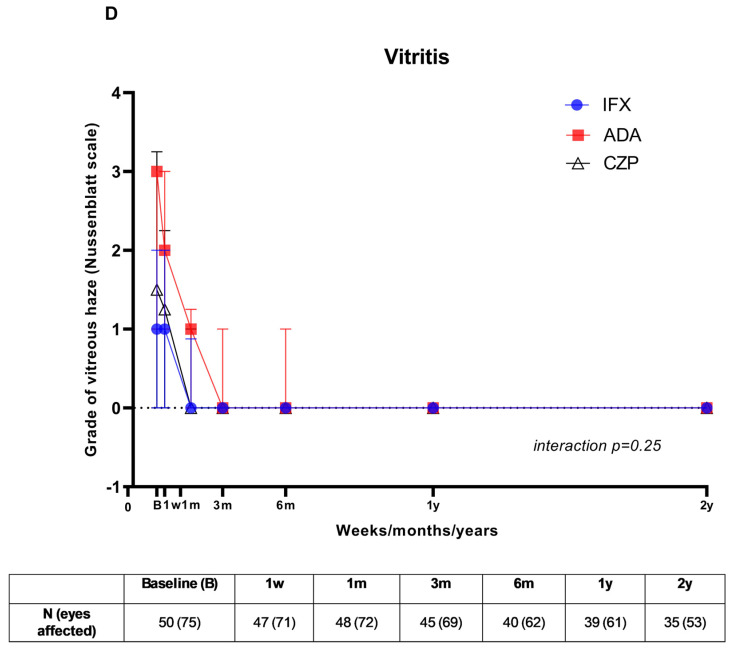
Rapid and maintained improvement following the onset of ADA, IFX and CZP. (**A**) Macular thickness measured by OCT (mean ± SD); (**B**) best-corrected visual acuity (BCVA) (mean ± SD); (**C**) anterior chamber (AC) cells—Tyndall (median (IQR)); (**D**) vitritis (median (IQR)).

After 2 years of follow-up, ocular variables evaluated in each group showed significant improvement compared to the baseline visit at the start of the administration of the different drugs (Figure 1). Macular thickness measured by OCT significantly decreased progressively, with no difference found between the three drugs after follow-up (ADA 264.76 ± 39.96, IFX 268.81 ± 32.73 and CZP 266 ± 44.18 μm; *p* = 0.94). Moreover, changes in OCT over time were similar across the three treatment groups (interaction *p* = 0.26) (Figure 1A). Likewise, a significant increase in BCVA was observed in the three treatment groups, with no differences between them at 2-year follow-up (ADA 0.74 ± 0.28, IFX 0.63 ± 0.36 and CZP 0.82 ± 0.16; *p* = 0.63), and the evolution of BCVA was similar in the three groups during the follow-up (interaction *p* = 0.12). (Figure 1B). Regarding the parameters that directly measure ocular inflammation, we observed a significant decrease in anterior chamber cells in the ADA (0 (0)), IFX (0 (0)) and CZP groups (0 (0)) (*p* = 0.63) at 2 years of follow-up. Remarkably, although interaction *p* between groups during follow-up was statistically significant (*p* = 0.03), no differences were observed at 2 years. (Figure 1C). Positive data were also observed in vitritis measurement in all patients (*p* = 0.64), with no significant changes in vitritis over time between groups (interaction *p* = 0.25) (Figure 1D).

Concerning the median follow-up of patients, no differences were observed between the three treatment groups (ADA 24 (3–36), IFX 24 (3–36) and CZP 30 (24–60) months; *p =* 0.12). Additionally, no significant differences were observed in terms of adverse events or remission of ocular activity (Table 1). No adverse events were observed in the CZP group. However, one patient treated with ADA presented with bacteremia in the context of pyelonephritis, and another patient experienced a mild infusional reaction in the IFX group. The biologic drug was discontinued in seven patients with ADA, eight IFX patients and two CZP patients, with no statistically differences found. Treatment was discontinued in two patients in the CZP group and two patients in the IFX group due to sustained remission. Ineffectiveness was the reason for drug discontinuation in seven patients treated with ADA and in six IFX patients.

## 4. Discussion

The management of refractory CME in uveitis related to inflammatory diseases currently remains a challenge. In most non-infectious uveitis, including BD, the amplification of the innate immune response and differentiation of CD4+ T lymphocytes to Th1/Th17 lymphocytes stimulate the production of proinflammatory interleukins (TNF-alpha, IL-6 or IL-17) that lead to blood–retina barrier breakdown and the accumulation of fluid in the macular space [37,38].

In recent decades, the development of drugs targeting TNF-alpha has been one of the main keys in the treatment of multiple inflammatory processes, including uveitis. However, only ADA is approved by the EMA and FDA for the treatment of non-infectious uveitis [15,16,17]. The use of other anti-TNF-alpha drugs such as GOL or CZP are not yet indicated despite having demonstrated effectiveness in real clinical practice and are usually reserved for patients in which ADA is ineffective or contraindicated. Regarding ADA and IFX, EULAR guidelines for the treatment of BD domains propose their use in patients with insufficient responses to conventional IS or in case of severe involvement that may lead to vision loss, but there is no recommendation with other anti-TNF-alpha drugs [39].

In the best of our knowledge, we present the first comparative study of three anti-TNF-alpha drugs (ADA, IFX and CZP) in the treatment of patients with CME due to BD uveitis. From a previously established cohort of 177 patients with BD uveitis treated with anti-TNF [26] and from a series of 80 refractory patients with immune-mediated inflammatory disease (IMID) uveitis treated with CZP [29], we selected those presenting CME-related BD uveitis at baseline time (25, 15 and 10 patients treated with ADA, IFX and CZP, respectively). After 2 years of treatment, CME resolution was achieved in three groups, in 75%, 60% and 70% in the ADA, IFX and CZP groups, respectively (*p* = 0.66). Improvements were also observed in the other effectiveness ocular outcomes evaluated, such as visual acuity (BCVA), presence of anterior chamber cells and vitritis in all groups.

As previously mentioned, there are several studies that have demonstrated the effectiveness and safety of anti-TNF-alpha drugs in the treatment of refractory BD uveitis, mainly patients treated with ADA and IFX [20,21,22,23,24,25,40,41,42]. Nevertheless, studies including CZP and more specifically focusing on the refractory treatment of BD-related CME are very scarce. In this regard, Tosi et al. reported five patients with BD uveitis treated with CZP, who had previously received another IS, with significative improvement in the first year. Three of them had previously received another anti-TNF [43]. In the same way, a Spanish observational study conducted by Llorenç et al. reported the complete remission of patients with chronic uveitis treated with CZP and prior failure to anti-TNF-α drugs [44]. Another study conducted by Lopalco et al. describes successful treatment with CZP in five BD patients with ocular involvement refractory to conventional IS and anti-TNF-alpha drugs [27].

An important consideration is the approach of sequential therapy in refractory CME patients. Drugs targeting TNF-alpha have shown extensive evidence in a large group of immune-mediated inflammatory diseases. Rheumatoid arthritis was one of the first indications in patients with insufficient responses to conventional treatment with synthetic DMARDs. However, for the last decade, physicians have been considering how to proceed when biological drugs primarily fail [45]. In general, in patients with rheumatoid arthritis who have experienced primary failure to an anti-TNF-alpha drug, switching to a different therapeutic target (IL-6, CTLA-4, JAK) is postulated to be more effective than cycling to another anti-TNF-alfa [46,47,48,49]. However, in our study, we present data from patients who had previously experienced a poor response to treatment with anti-TNF-alpha drugs (especially ADA and IFX) and who, after the administration of a new anti-TNF-alpha drug, CZP, achieved complete responses and even remission of ocular involvement. In this regard, we maintain that cycling may be an effective therapeutic strategy in BD patients with ocular manifestations.

Infections are the most frequent side effects of anti-TNF-alpha drugs. However, in our study, only one patient treated with ADA experienced a serious infection (bacteremia secondary to pyelonephritis). Other relatively frequent adverse effects are infusional reactions or neoplasms. Only one IFX patient had a mild infusional reaction that did not cause the discontinuation of treatment.

However, considering the unequal sample size, it will not be possible to assess the safety of any therapeutic regime used in the study.

There are several limitations inherent in our study, mainly due to its observational nature, the lack of a control group, and the relatively small number of patients. For these reasons, more randomized controlled trials comparing conventional immunosuppressive drugs and other biological therapies are needed. However, it is challenging to carry out studies on BD refractory uveitis and biologic therapies, especially because of the single approval of ADA in the treatment of this pathology. Therefore, it is likely that in the future, valuable information will come from observational multicenter studies such as ours.

We have taken into account that our sample size was not large. We have not performed a paired Student’s *t*-test, but rather a mixed models analysis. For this reason, conducting a Welch’s test would not be applicable in this case. However, it is known that linear mixed models can be robust, even under small sample size conditions [50].

In conclusion, the results of our study suggest that ADA, IFX and CZP are safe and effective treatment for patients with CME due to BD. In addition, CZP demonstrates effectiveness in patients with insufficient responses to other anti-TNF-alpha drugs such as ADA or IFX, so a cycling strategy could be an appropriate alternative in these cases.

## Figures and Tables

**Table 1:** Main general features of a series of 50 patients with cystoid macular edema (CME) due to Behçet’s disease (BD) uveitis treated with adalimumab (ADA), infliximab (IFX) and certolizumab pegol (CZP).

	ADA Group *n* = 24	IFX Group *n* = 15	CZP Group *n* = 10	*p*
Age, mean (SD) years	41 (11)	38 (9)	36 (8)	0.42
Sex, men/women, *n*/*n*	12/12	7/8	3/7	0.65
HLA-B51-positive, *n* (%)	19 (79)	10 (67)	4 (40)	0.07
Duration of uveitis before anti-TNF, median (IQR) months	27 (12–60)	15 (8–60)	72 (36–120)	0.03
Number of eyes with CME, *n* (%)				
Unilateral	10 (42)	9 (60)	3 (30)	0.33
Bilateral	14 (58)	6 (40)	7 (70)	
Ocular features at the time of anti-TNF/anti-IL6 therapy onset				
AC cells (Tyndall), median (IQR)	2 (1–3)	1 (0–1.5)	1 (0–2)	0.099
Vitritis, median (IQR)	2.75 (1.75–3)	1 (0–2)	1 (0–2)	0.025
BCVA, median (SD)	0.4 (0.2–0.6)	0.3 (0.2–0.5)	0.20 (0.25)	0.19
Macular Thickness, mean (SD)	437 (117)	483 (126)	381 (96)	0.084
Pattern of uveitis, *n* (%)				
Anterior	24 (100)	15 (100)	8 (80)	0.038
Posterior	4 (83)	5 (33)	3 (33)	0.43
Panuveitis	20 (83)	10 (67)	4 (40)	0.051
Previous treatment before anti-TNF therapy onset, *n* (%)				
Intravenous pulses of mehtylprednisolone	13 (54)	9 (60)	5 (50)	0.87
Cyclosporine A	22 (92)	11 (73)	6 (60)	0.07
Azathioprine	14 (58)	8 (53)	4 (40)	0.70
Methotrexate	12 (50)	8 (53)	2(20)	0.24
Adalimumab	0 (0)	0 (0)	7 (70)	<0.001
Infliximab	0 (0)	0 (0)	7 (70)	<0.001
Etanercept	0 (0)	0 (0)	1 (10)	<0.001
Golimumab	0 (0)	0 (0)	2 (20)	<0.001
Prednisone dose at anti-TNF onset, median (IQR), mg/d	45 (25–60)	30 (20–60)	9 (6–20)	0.051
Combined treatment at anti-TNF/anti-IL6 onset, *n* (%)				
Azathioprine	2 (13)	3 (20)	2 (20)	0.68
Cyclosporine A	10 (42)	5 (33)	1 (10)	0.25
Methotrexate	2 (8)	2 (13)	4 (40)	0.11
Follow-up on anti-TNF/anti-IL6 therapy, median (IQR), months	24 (18–45)	24 (3–36)	30 (24–60)	0.12
CME remission, *n* (%)	18 (75)	9 (60)	7 (70)	0.66
Drug withdrawal, *n* (%)	8 (32)	8 (53)	2 (20)	0.22
Side effects/toxicity, *n* (%)	1 (4)	1 (7)	0 (0)	0.51

*p* was the comparison between the adalimumab group, infliximab group and certolizumab pegol group. *p* < 0.05 was considered statistically significant.

## Data Availability

Data are contained within the article.
